# Environmental Injustice and Industrial Chicken Farming in Maryland

**DOI:** 10.3390/ijerph182111039

**Published:** 2021-10-20

**Authors:** Jonathan Hall, Joseph Galarraga, Isabelle Berman, Camryn Edwards, Niya Khanjar, Lucy Kavi, Rianna Murray, Kristen Burwell-Naney, Chengsheng Jiang, Sacoby Wilson

**Affiliations:** School of Public Health, College Park, University of Maryland, 4200 Valley Dr, College Park, MD 20742, USA; jhall123@umd.edu (J.H.); isabellerb15@gmail.com (I.B.); camryn.v.edwards@gmail.com (C.E.); nkhanjar@umd.edu (N.K.); lkavi@umd.edu (L.K.); rmurray@umd.edu (R.M.); kbnaney@gmail.com (K.B.-N.); cjiang89@umd.edu (C.J.); swilson2@umd.edu (S.W.)

**Keywords:** environmental justice, confined animal feeding operations, people of color, disparities, poverty

## Abstract

Maryland’s growing chicken industry, including concentrated animal feeding operations (CAFOs) and meat processing plants, raises a number of concerns regarding public health and environmental justice. Using hot spot analysis, we analyzed the totality of Maryland’s CAFOs and meat processing plants and those restricted to the Eastern Shore to assess whether communities of color and/or low socioeconomic status communities disproportionately hosted these types of facilities at the census tract level. We used zero-inflated regression modeling to determine the strength of the associations between environmental justice variables and the location of CAFOs and meatpacking facilities at the State level and on the Eastern Shore. Hot spot analyses demonstrated that CAFO hot spots on the Eastern Shore were located in counties with some of the lowest wealth in the State, including the lowest ranking county—Somerset. Zero-inflated regression models demonstrated that increases in median household income across the state were associated with a 0.04-unit reduction in CAFOs. For every unit increase in the percentage of people of color (POC), there was a 0.02-unit increase in meat processing facilities across the state. The distribution of CAFOs and meat processing plants across Maryland may contribute to poor health outcomes in areas affected by such production, and contribute to health disparities and health inequity.

## 1. Introduction

Rural farming has changed drastically in the last fifty years, with the influx of corporate entities into the agriculture and livestock business and the decline of small-scale, family-owned farms becoming the norm [1]. The increased industrialization of agricultural operations has resulted in an overall decrease in the number of farms, but an increase in the size. For example, the number of farms in the United States fell from approximately 6.8 million in 1935 to 2.1 million in 2002, and the average farm size grew from 154.8 acres to 434 in the same time frame [2]. The number of animals raised at industrial production facilities increased by nearly 246% between 1982 and 2002 while the total number of livestock raised in the year 2000 was equal to that of the previous 80 years [2].

The practice of concentrating farm animals into limited land spaces has led to the characterization of these facilities as animal feeding operations (AFOs) [3]. A facility is characterized as a concentrated animal feeding operation (CAFO) when it confines animals for at least 45 days within a year, no grass or other vegetation grows in the confinement area, and contains more than 100 animal units [4]. At CAFOs, animals are held throughout their lives in indoor stalls until they are transported to processing plants for slaughter.

Due to the concentration of animals and the density of industrial farms, these operations pose threats to environmental and human health. For example, thousands of pounds of harmful emissions including volatile organic compounds (VOCs), ammonia, nitrogen, carbon dioxide, methane, particulate matter (PM), and heavy metals are emitted from CAFOs on an annual basis [5,6,7]. For these reasons, the U.S. Environmental Protection Agency (US EPA) has classified CAFOs as point source polluters under the Clean Water Act [8]. Consequently, CAFOs must receive permits through the Clean Water Act’s National Pollution Discharge Elimination System (NPDES) to control for manure, nutrient, and waste runoff into waters [9].

These operations pose health problems for individuals who live nearby. For example, local residents face exposure to heavy metals, fertilizers, pesticides, and 355 million tons of animal waste emitted annually that humans may be exposed to via the air and runoff waste in groundwater and surface water [7,10,11]. Researchers found that distance from a CAFO or multiple CAFOs is a key to understanding weekly atmospheric ammonia levels [12] and the closer the populace was to the hog CAFO, the more intense the exposure [13].

Additionally, chemicals found in heavily concentrated manure produced from CAFOs are responsible for 37% of national methane emissions and 65% of national nitrous oxide gases released annually [14]. PM_10_ accounts for one-third of the total emissions released from CAFOs, and excess amounts of arsenic emitted into water sources are linked to health issues like bladder, lung, skin, nasal, liver, kidney, and prostate cancer [15]. CAFOs generate excess nitrogen, above the federal standard, with chicken farms making up 60% of operations exceeding the acceptable nitrogen levels [2]. Overexposure to nitrates is associated with methemoglobinemia, hypertension, infant mortality, goiter, stomach cancer, thyroid disorder, cytogenetic defects, and birth defects [16].

The poor air quality surrounding CAFOs differentially impacts nearby residents and on-site workers who have higher exposures than the general population in occupational settings [17]. High rates of respiratory and gastrointestinal problems, as well as irritation in the eyes and throat from airborne emissions, have been recorded by communities in close proximity to swine CAFOs, with the highest rate being experienced by employees of the operation [18]. More recent studies show that 25% of employees of CAFOs report respiratory problems thought to be caused by exposure to endotoxin, a family of Gram-negative bacteria membrane lipopolysaccharide fragments that are mainly found in environments that have high exposure to organic dusts, that are known to be present in high concentrations specifically in chicken CAFOs [19,20,21]. A study conducted in North Carolina found that children of CAFO workers are more than twice as likely to contract methicillin-resistant Staphylococcus aureus (MRSA) than children in households with no contact with these operations [22]. Similarly, the prevalence of Staphylococcus aureus and MRSA in CAFO workers has been found to be higher than in the general U.S. population [23].

Animal agriculture has been shown to differentially burden low-income communities and communities of color [17,24,25,26]. A study in Mississippi found hog operations were located in areas with high percentages of low-income, African American residents [27]. North Carolina studies have found that low-income communities and communities of color were more likely to be located near a CAFO than their wealthier, White counterparts [20,28]. A 2015 study determined that communities with high percentages of Hispanic individuals are disproportionately burdened by CAFOs in Ohio [29]. Furthermore, various North Carolina studies have determined that vulnerable populations are disproportionately burdened by poor air quality and high ammonia levels as a result of close proximity to CAFOs [13,30].

From 2011 to 2018, the number of CAFOs in the state of Maryland rose from 150 to 573 (a 282% increase), suggesting that it is a continuously growing industry. In the same time frame, there was a 46.5% increase in egg production in Maryland [31]. To this point, no study has examined whether or not there are environmental justice issues associated with chicken farming in the state of Maryland. This paper explores potential disparities in the distribution of chicken farms in Maryland along sociodemographic lines, and discusses the implications on human health and quality of life in the region.

## 2. Materials and Methods

### 2.1. Data Sources

We obtained geocoded data for concentrated animal feeding operations (CAFOs) from the Maryland Department of Environment’s (MDE) AFO permit database, which provides both CAFO and AFO data for different animal feeding industries. The Topologically Integrated Geographic Encoding and Referencing (TIGER) file, a census tract boundary file, was downloaded from the MDE’s 2010 Maryland Census Tract boundary dataset [32]. This was then spatially joined with sociodemographic features from the 2018 Census Bureau American Community Survey (ACS) 5-year estimates data obtained from the Census [33]. The sociodemographics used for analyses were: (1) percentage people of color; (2) percentage under 18; (3) percentage below poverty; (4) percentage individuals without a high school (HS) diploma over age 25; (5) percentage below poverty who are under 18; (6) median household income; (7) per capita income; (8) percentage of home ownership; and (9) percentage of homes built before 1949. Housing pre-1949 was chosen to represent suburbanization based on historical trends in development [34]. Similar variables have been used in previous environmental justice research to represent race/ethnicity and socioeconomic status [18,20,27]. Due to the inconsistent reliability of ACS census data, we excluded any census tracts where the coefficient of variation was greater than 40, which the census bureau considers unreliable [35]. This reduced the total number of populated census tracts in the analysis from 1394 down to 1309 total census tracts. Additionally, data related to poverty, education, and housing pre-1949 were removed from the analysis due to its particular unreliability.

We obtained the address and primary activity for all meat processing facilities in the United States from the US Department of Agriculture’s (USDA) Meat, Poultry, Egg Inspection Directory [36]. The address of each facility was geocoded and then mapped as individual points in ArcGIS.

### 2.2. Statistical Analyses

The first stage of analysis required identifying CAFO host and non-CAFO host census tracts. A “CAFO host tract” was defined as a census tract that contained 1 or more CAFOs, whereas a “non-CAFO host tract” was defined as a census tract that did not contain a CAFO within its boundaries. The same definitions of host and non-host census tracts were used for meat processing facilities. These census tract categorizations were then stratified by majority “rural” or “urban” designations based upon urban and rural housing unit data from the 2010 Census. Census tracts with more than 50% urban housing units were considered “urban” and those with more than 50% rural housing units were designated as “rural”. ArcGIS 10.7 (ESRI, Redlands, CA, USA) was used to find these census tract classifications, and these were then analyzed alongside sociodemographic statistics to draw correlations between presence/distance from CAFO and meat processing sites versus socioeconomic status and race/ethnicity.

A hotspot analysis using the “Optimized Hot Spot Analysis” test was performed on CAFOs and meat processing facilities in Maryland in order to find areas with large concentrations of CAFOs and meat processing plants. For CAFOs a distance band of 25,284.14 m was calculated, while one of 12,256 m was calculated for meat processing facilities. Because of the concentration of CAFOs on the Eastern Shore, a secondary “Optimized Hot Spot Analysis” test was used to further investigate CAFO concentrations in this region. The distance band used for this analysis was 19,887.27 m. The average sociodemographics of areas determined to be CAFO/meat processing hotspots or cold spots were found in order to understand the primary population characteristics impacted by the animal agriculture industry. A comparison between the mean differences between both CAFO and meat processing hotspots was completed using the study variables.

To build upon the results from the hotspot analysis and identify statistically significant relationships, statistical tests were run to further understand the relationships between the chicken industry and socioeconomic indicators: Exploratory and Ordinary Least Squares (OLS) regression were performed to determine if there was a statistically significant relationship between sociodemographic variables in census tracts versus the presence of CAFOs and related sites using ArcGIS. Additionally, multivariate regression tests were performed in PAST 4.03 (University of Oslo, Oslo, Norway) to determine if there is a relationship between the presence of the chosen demographic, income, and education variables versus the number of CAFOs/meat processing facilities. However, due to the number of census tracts that contained zero CAFOs or meat processing facilities, a zero-inflation regression model was used instead. This was performed because the large frequency of zero CAFO/meat processing facility census tracts skewed the model, causing it to not be normally distributed due to the excessive number of zeroes. A separate zero-inflation regression was also used to assess the relationship between the sociodemographics and CAFOs on the Eastern Shore, as this area was identified as a chicken CAFO hot spot. The zero-inflation model was performed in RStudio (R Consortium, Boston, MA, USA). In order for the test to be completed, the median household income variable was scaled down by 1000. Finally, the regressions were offset using total population per census tract in order to adjust for the differences in population between urban and rural areas.

## 3. Results

**Figure 1:**
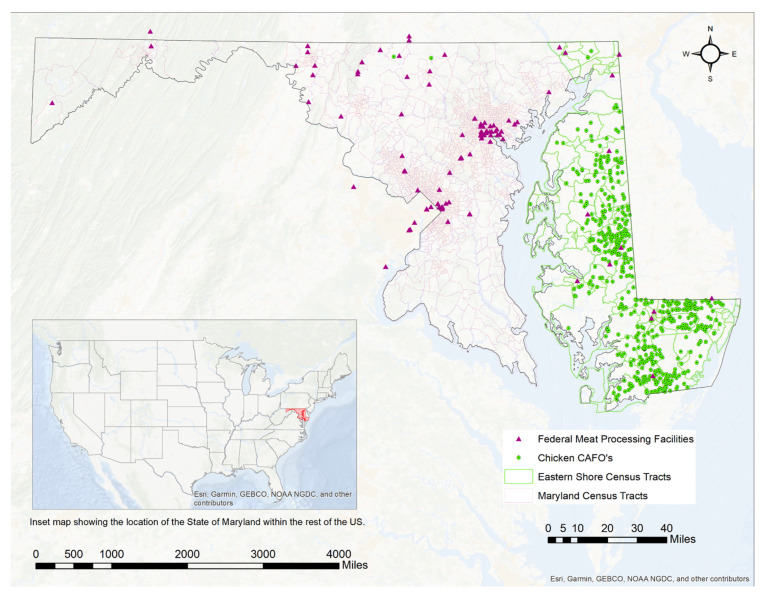
Locations of CAFOs and Federal Meat Processing Facilities in Maryland.

Table 1 shows that census tracts that host CAFOs in more urban areas feature a 27.5% POC population vs. those in rural areas (15.3% POC). Additionally, these census tracts tended to have lower median household incomes and levels of homeownership in comparison to rural areas with CAFOs. However, there were 35 rural census tracts that hosted CAFOs, while there were only 12 urban census tracts that were hosts (See Figure 1).

**Figure 2:**
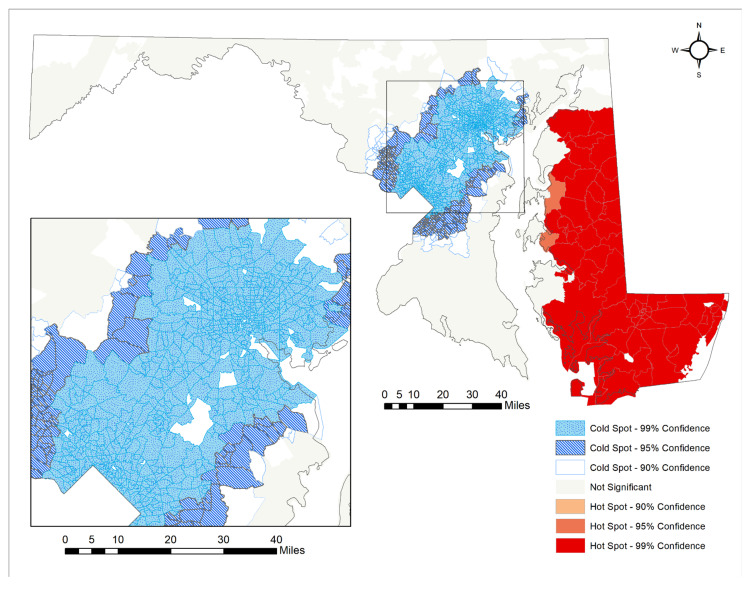
Optimized Hot Spot Analysis for Chicken CAFOs in Maryland.

**Figure 3:**
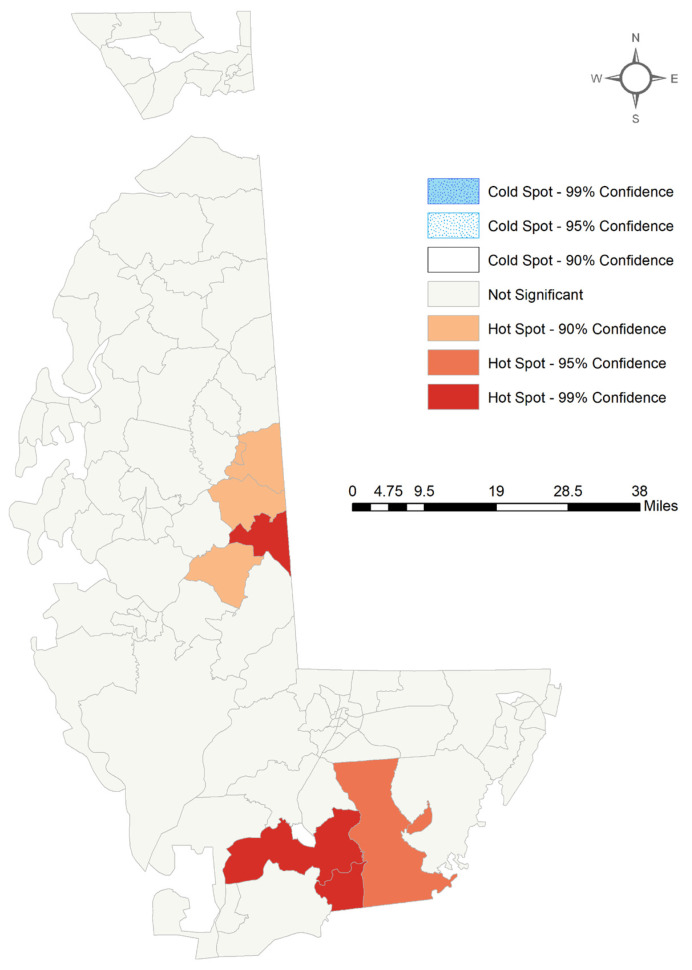
Optimized Hotspot Analysis for Chicken CAFOs on the Eastern Shore of Maryland.

In Figure 2, the primary hotspots of CAFOs are centered around the Eastern Shore and the Delaware border, whereas the CAFO coldspots are located in the Baltimore–Washington Metropolitan area. The meat processing facility hot spots are generally more dispersed throughout the state, with a larger concentration of statistically significant hotspots occurring in and around Baltimore. However, due to the distribution of meat processing facilities throughout the state, there are no cold spots of meat processing facilities. When looking at the Eastern Shore of Maryland exclusively, the southern part of the Eastern Shore, around Dorchester and Wicomico Counties, is identified as a concentration of CAFO hotspots as seen in Figure 3. The hotspot classifications were used in Table 1 and Table 2 as categories for highlighting the differences in means between hot spots and cold spots of CAFOs and meat processing facilities.

Figure 1 shows the location of chicken CAFOs and federal meat processing plants in the state of Maryland. Chicken CAFOs are shown to be concentrated on the Eastern Shore of the state, with very few outliers in other regions. meat processing plants, conversely, are primarily located in more urban areas, specifically in and around the city of Baltimore. However, there are other meat processing plants located throughout the western part of Maryland. There is very little overlap between hotspots for chicken CAFOs and the location of meat processing plants.

**Figure 4:**
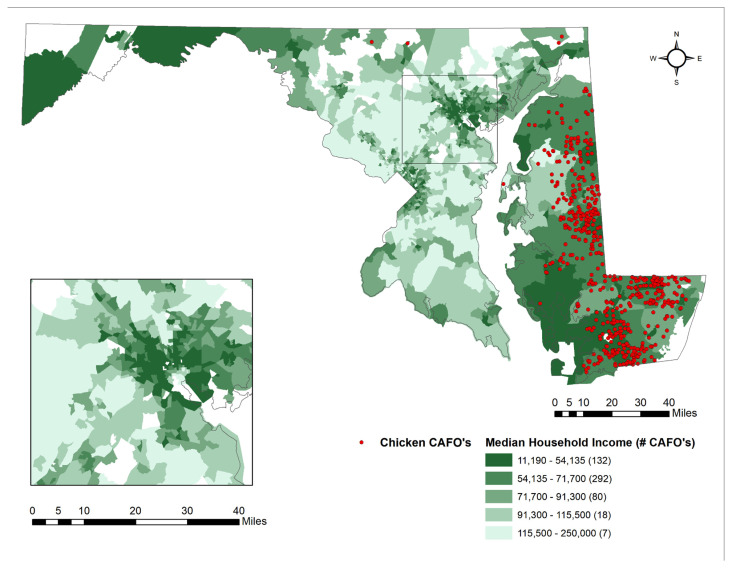
Chicken CAFOs by Quintiles of % Population of Median Household Income.

Figure 4 demonstrates the locations of CAFOs and their colocation to populations of people as stratified by median household income. CAFOs are abundant on the Eastern Shore of Maryland, which also has many areas with a large percentage of people with lower median household incomes.

**Figure 5:**
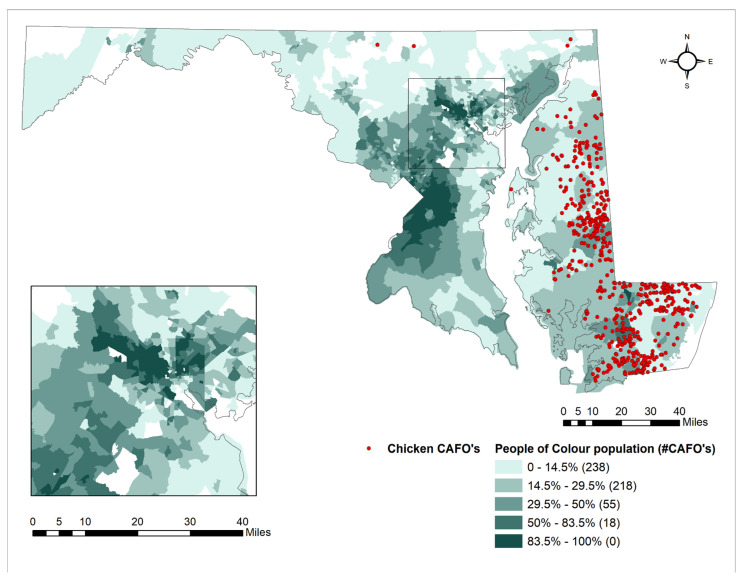
Chicken CAFOs by Quintiles of % Population POC.

The largest concentrations of people of color tend to be in the Baltimore area which showed a spatial relationship to the presence of CAFO coldspots (Figure 5). On the other hand, there is a comparatively smaller population of people of color on the Eastern Shore where large CAFO hotspots exist (Table 2). However, the percentage of people with low median household incomes tended to be high in both the Baltimore and the Eastern Shore areas. When comparing this to the CAFO and meat processing facility locations in Figure 1, there appears to be a tendency for CAFOs and meat processing facilities to be located in areas with low median household incomes (see Figure 5).

With regards to race/ethnicity, meat processing facilities appear to be located in areas of higher POC, in contrast to the distribution of CAFOs which appears to be in smaller POC percentage census tracts. The percentage of homeownership, as opposed to rented homes, is slightly lower in CAFO hotspots than in CAFO coldspots (Table 2).

However, the median household income of census tracts in these hotspots was inversely related to the presence of a CAFO hotspot, with the highest median household income areas being statistically significant coldspots or statistically insignificant census tracts, which tended to be urban or suburban areas. The coldspots, however, included the areas around Baltimore which had much higher than average median household incomes than inside Baltimore.

Table 3 displays the results of the zero-inflated Poisson regression for chicken CAFOs. This analysis produced a number of statistically significant results. Increases in median household income across the state were associated with a 0.04-unit reduction in CAFOs. As the percentage of homeownership increased, there was a 0.05-unit increase in CAFOs.

Table 4 displays the results of the zero-inflated Poisson regression for meat processing facilities. This analysis shows that for every unit increase in the percentage of POC, there is a 0.02-unit increase in meat processing facilities across the state.

Based upon the results of the hotspot analysis that demonstrated statistically significant clustering of chicken CAFOs in Maryland’s Eastern Shore, a zero-inflated Poisson regression was used to assess relationships between sociodemographics and the presence of CAFOs in the region. Prevalence ratios were also used to determine if the prevalence of CAFOs was modified by sociodemographics. The zero-inflated Poisson regression model shows that for every unit increase in median household income, there is a 0.04-unit reduction in CAFOs on the Eastern Shore (see Table 5). Increases in % homeownership were associated with a 0.05-unit increase in CAFOs on the Eastern Shore.

## 4. Discussion

The goal of these analyses was to examine the relationship between sociodemographic characteristics and presence to chicken CAFOs and meat processing plants in the state of Maryland, specifically assessing communities of color and low-income communities to determine if they are disproportionately burdened by industrial chicken farming and processing plants. Results from the zero-inflated Poisson regression model (Table 3) show a positive relationship between POC and chicken CAFOs statewide, yet this association is not statistically significant at a *p*-value of 0.05. This finding is also consistent with work performed in similar studies, which demonstrate a higher percentage of African American residents in communities located near CAFOs [20,26,27,28]. We found that the largest clusters of CAFOs are located on the Eastern Shore. Table 2 demonstrates that 25.2% of the population in CAFO hotspots are POC. Additionally, this region features some of the lowest median household incomes in the state (Figure 4). This is a significant finding because socioeconomic status appears to have a strong correlation with the location of chicken CAFOs, so much so that economically depressed areas seem to be targets for CAFO operations [37]. The trend held true beyond the state level and extended to Maryland’s CAFO hot spot on the Eastern Shore. Zero-inflated Poisson regression restricted only to the Eastern Shore also demonstrated that low median household incomes were associated with chicken CAFOs. This is detrimental to the wellbeing of residents living within close proximity to CAFOs due to the health implications such as respiratory diseases and irritation of the eyes, nose, and throat [20,27]. Manure, antibiotics, and heavy metals are all produced by these animal farms and have the potential to seep into nearby water sources or contribute to pollution in the area [7].

Statistical analyses demonstrated that chicken CAFOs are unequally distributed throughout the state, with a disproportionate amount located near low-income communities. This finding is consistent with work performed in previous studies, which determined that CAFOs in North Carolina and Mississippi have the tendency to be located near low-income communities [27]. On the Eastern Shore, Bernhardt et al. (2015) found that there were 438 chicken operations in Caroline, Dorchester, Somerset, Wicomico, and Worcester counties in 2013, totaling 243,891,955 animals [38]. The high concentration of CAFOs in the region is an environmental justice concern, given these five counties’ SES. All five fall below the state’s 2014 median household of USD 74,149 (Caroline—USD 55,605, Dorchester—USD 45,628, Somerset—USD 36,716, Wicomico—USD 52,301, and Worcester—USD 58,820). All five are also above the state average of people below the poverty level (10%)—15.3% in Caroline, 16.9% in Dorchester, 23.7% in Somerset, 17.4% in Wicomico, and 11.1% in Worcester. Only Somerset, however, has a higher African American population (42.3%) and a lower White population (53.5%) than the State (29.4% Black and 58.2% White, respectively) [39]. This study fills an important gap in the literature with respect to assessing environmental justice concerns related to Maryland’s chicken industry.

Studies have shown that CAFOs are usually developed in existing communities of color and low-income communities, instead of the CAFO attracting these populations [26]. Individuals that have the financial means to leave the area following the development of a CAFO usually do, leaving residents of low SES to suffer the environmental and public health consequences of living near a CAFO [40].

This analysis showed a positive relationship between the percentage of homeownership and the presence of CAFOs, indicating that homeownership may not be an accurate indicator of socioeconomic status, but rather an indicator of rurality or urbanity. Homeownership is more common in rural areas [41], which would help explain why homeownership is associated with an increased presence of CAFOs, which primarily affect rural populations [42].

In contrast to CAFOs, meat-packing plants were shown to be distributed more evenly across the state. Numerous plants were located in Baltimore City (Figure 3). Air quality in Baltimore City has already caused concern regarding residents’ health and well-being [43], and the high concentration of meat-packing plants and increasing industrialization have the potential to contribute to the declining public health of the city [44]. Statistical analysis from Table 4 demonstrates comparable results to Table 2, showing that meat processing facilities, like chicken CAFOs, are disproportionately located near low-income communities of color. This is consistent with studies performed in other areas of the United States, specifically the Midwest, which determined areas containing meat processing facilities to have high percentages of immigrants and low-income families [44].

Table 4 shows higher percentages of homeownership associated with meat processing facilities, similar to CAFOs. As stated previously, this distinction is consistent with studies showing higher rates of homeownership in rural areas [41]. Although there are more meat processing facilities in urban areas than there are CAFOs, there are still enough in areas of high homeownership to create an overall positive correlation between homeownership and meat processing facilities.

Existing research regarding demographics surrounding meat processing plants shows an overwhelming correlation between the location of meat processing plants and areas with high immigrant populations [44,45,46,47]. This correlation is consistent with the analysis shown in Table 3, indicating increases in POC associated with a unit increase in meat processing facilities, as 76.5% of immigrants in the state of Maryland are POC [39]. Additionally, in the state of Maryland meat processing facilities are concentrated in Washington, DC and Baltimore City which both have immigrant populations that make up approximately 13–15% of the total population [48]. Future research regarding meat-packing facilities in the state should investigate the specific meat-packing plants that exist in Baltimore City and how their presence in and around the city impacts the health of residents.

### 4.1. Limitations

This study has several limitations. Many of the demographic variables are proxies to try to assess urban/suburban/rural relationships, and therefore cannot offer more precise relationships. For example, the large percentage of houses built before 1949 in Baltimore and in the Eastern Shore is due to these areas’ histories as urban and agricultural centers, respectively. The mass suburbanization of America occurred after World War II, meaning the majority of suburban homes were built following 1949 [34]. Suburbanization has also led to larger concentrations of POC living in the inner cities, and despite a large growth of POC moving to the suburbs, large areas of suburbia remain majority White [49]. This proxy measure cannot fully capture the dynamics related to suburbanization, and therefore is a limitation in the results.

There were several census tracts that had to be disqualified from this study due to unreliable ACS data. Because of this, it is difficult to assess how these tracts may affect the overall results for the state of Maryland. Additionally, there were several variables that similarly were excluded from the analyses due to unreliable ACS data due to high coefficients of variation. Because of this, it was impossible to measure associations between the presence of CAFOs, meatpacking facilities, and poverty—which could illuminate the discussion regarding the association between socioeconomic status and the presence of industrial chicken farming.

Performing these analyses at a lower level of analyses could have produced more precise effects. Other investigations of environmental justice variables in relation to CAFOs have been conducted at the block group level instead of the census tract level [20,27]. Aggregation of data at a higher level of analysis produces more heterogeneous data, which can mask effects occurring on a smaller scale [50].

### 4.2. Implications and Recommendations

The results of this investigation have implications for both policymaking and future research studies. Because this work has demonstrated that CAFOs and meat processing facilities disproportionately burden areas with higher concentrations of POC and low socioeconomic status communities in Maryland, more policy provisions should be implemented to protect these populations. Public comment periods where citizens can give input on a federal level are a way to temporarily deal with problems due to CAFOs [19,51]. In addition, the Maryland Department of the Environment requires the inclusion of the distance to and water quality status of the nearest body of water and potential outdoor air quality risks in the Comprehensive Nutrient Management Plan (CNMP), which is necessary to get a permit to build a CAFO [51]. While MDE does have several recommended stopgaps in place regarding the establishment of new CAFOs, this study suggests that Maryland’s chicken industry policy needs to employ an environmental justice lens. Environmental justice-oriented solutions include policy that promotes community empowerment; building infrastructure that supports sustainable communities; enhancing community-based pollution prevention strategies; and creating community-based sustainable development [52]. Essentially, policy solutions should be holistic in their approach to providing protection for environmental justice communities through initiatives that empower communities rather than merely being prescriptive. Policy that adopts the environmental justice framework also focuses on prevention as opposed to treatment [52]. Therefore, a public health approach to policy that prioritizes the prevention of exposure to the deleterious byproducts of CAFOs and meat processing plants is essential to the regulation of these industries.

This study produced notable results regarding the unequal distribution of CAFOs and meat processing plants amongst communities of color and low socioeconomic status in Maryland. Other research should build upon these findings using cohort studies in order to further analyze the relationships between environmental justice communities and hazards associated with the chicken industry in Maryland. The present study relied on an ecological design. Future investigations should examine the presence of contaminants associated with the chicken industry, such as ammonia or nitrate, in environmental justice communities located near such sites in Maryland in order to measure actual exposure levels [12,53]. Previous research that could provide guidance to future work in this area includes Mirabelli’s (2007) cohort designs that used questionnaires to ask participants about their exposure history to agents that may exacerbate asthma [54]. Other research on agricultural contaminants has included biological sampling from a cohort of livestock workers to demonstrate exposure to antibiotic-resistant *Staphylococcus aureus* [55]. A biological sampling of nearby water supplies or residents’ homes and properties could provide a better understanding of exposures related to industrial chicken farming.

A dearth of pertinent data proved to be a challenge to the present investigation. Passing the Maryland Community Healthy Air Act would facilitate better data collection and further analysis. The proposed legislation creates a protocol for sampling, quantifying, and reporting various data related to large animal-feeding operations across Maryland [56]. These data—which include ammonia, particulate matter, and VOCs—would provide a better understanding of the types and amount of exposure that CAFO host communities experience, providing a stronger basis for policy and advocacy around environmental health and environmental justice.

## 5. Conclusions

As two of the leading food production businesses in the state [57], poultry CAFOs and meat processing facilities in Maryland must acknowledge the threats they pose to environmental and public health. Low socioeconomic status communities and communities of color are disproportionately burdened by chicken CAFOs and meat processing facilities across Maryland, making the state’s chicken industry an environmental justice concern. This study has produced results that suggest a need for further research into the lasting effects of industrial agriculture on community health. Efforts should be made by the state to reduce these disparate effects of chicken CAFOs and meat processing facilities through regulation and by providing protection for the communities that live and work in affected areas. Additionally, consistent data collection and reporting on environmental factors such as water and air quality is essential to monitoring and ensuring the environmental health of these areas. Future studies should continue to investigate Maryland’s chicken industry with respect to environmental justice concerns in order to provide further basis for policy to improve the environmental public health of affected communities throughout Maryland.

## Figures and Tables

**Table 1:** Distribution of Features/Sociodemographic Features, CAFO Host vs. Non-Host.

	CAFO Host		Non-Host	
	Urban	Rural	Urban	Rural
# of Census Tracts	12	35	1163	99
# of CAFOs	83	446	0	0
# of Meat Processing Facilities	2	4	70	9
% Population: People of Color	27.5%	15.3%	49.3%	16.2%
% Population Under 18	21.3%	20.4%	22.3%	20.7%
Median Household Income	64,059.83	66,626.29	86,313.37	101,345.24
% Homeownership	56.6%	67.7%	59.2%	76.8%

# of = “Number of”.

**Table 2:** CAFO and Meat Processing Hot/Coldspots vs. MD Sociodemographics.

Maryland	CAFO Hotspot	CAFO Coldspot	CAFO Insignificant	Meat Processing Hotspot	Meat Processing Coldspot	Meat Processing Insignificant	Maryland
# of Census Tracts	70	941	298	194	N/A	1115	1309
# of CAFOs	518	0	11	66	N/A	463	529
# of Meat Processing Facilities	8	56	14	31	N/A	47	78
% Population: People of Color	25.7%	54.7%	21.99%	59.8%	N/A	43.2%	45.7%
% Population Under 18	21.0%	22.2%	22.0%	21.7%	N/A	22.2%	22.1%
Median Household Income	61,060.87	88,492.63	87,149.13	55,797.43	N/A	92,100.06	86,719.84
% Homeownership	56.0%	58.9%	67.8%	43.9%	N/A	63.7%	60.7%

**Table 3:** Zero-inflated Poisson Regression Modeling for MD Chicken CAFOs.

Count Model	Estimate	Std. Error	*z* Value	Probability (>|*z*|)
Intercept	−6.86	0.46	−14.94	<2 × 10^−16^ ***
% Population: People of Color	8.75 × 10^−3^	5.03 × 10^−3^	1.739	0.082
% Population Under 18	−0.01	0.01	−0.88	0.379
Median Household Income (USD) (Scaled by 1000)	−0.04	4.07 × 10^−3^	−9.91	<2 × 10^−16^ ***
% Homeownership	0.05	6.09 × 10^−3^	8.87	<2 × 10^−16^ ***

Signif. codes: “***” *p* < 0.001.

**Table 4:** Zero-inflated Poisson Regression Modeling for MD Meat Processing Facilities.

Count Model-Meat Processing	Estimate	Std. Error	*z* Value	Pr (>|*z*|)
(Intercept)	−12.19	1.36	−9.00	<2 × 10^−16^ ***
% Population: People of Color	0.02	7.92 × 10^−3^	3.02	2.55 × 10^−3^ **
% Population Under 18	0.05	0.05	1.10	0.27
% Homeownership	0.02	0.02	1.35	0.18
Median Household Income (USD)	−8.23 × 10^−3^	0.01	−0.72	0.47

Signif. codes: “***” *p* < 0.001, “**” *p* < 0.01.

**Table 5:** Zero-inflated Poisson Regression Modeling for MD Chicken CAFOs in the Eastern Shore.

Count Model-Eastern Shore	Estimate	Std. Error	*z* Value	Pr (>|*z*|)
(Intercept)	−6.55	0.44	−14.90	<2 × 10^−16^ ***
% Population: People of Color	3.82 × 10^−3^	5.02 × 10^−3^	0.72	0.45
% Population Under 18	−0.01	0.01	−1.20	0.23
% Homeownership	0.05	5.96 × 10^−3^	8.52	<2 × 10^−16^ ***
Median Household Income (USD)	−0.04	3.90 × 10^−3^	−9.85	<2 × 10^−16^ ***

Signif. codes: “***” *p* < 0.001.

## Data Availability

Data for these analyses are available from the Maryland Department of Environment’s (MDE) AFO permit database, the Terrestrial Initiative in Global Environmental Research (TIGER), and the 2018 American Community Survey from the Census.

## References

[1] Dimitri C., Effland A., Conklin N. (2005). The 20th Century Transformation of U.S. Agriculture and Farm Policy.

[2] USDA—National Agricultural Statistics Service—Census of Agriculture. https://www.nass.usda.gov/AgCensus/.

[3] (2019). Concentrated Animal Feeding Operations. https://www.ncsl.org/research/agriculture-and-rural-development/concentrated-animal-feeding-operations.aspx.

[4] Animal Feeding Operations|NRCS. https://www.nrcs.usda.gov/wps/portal/nrcs/main/national/plantsanimals/livestock/afo/.

[5] Yuan B., Coggon M.M., Koss A.R., Warneke C., Eilerman S., Peischl J., Aikin K.C., Ryerson T.B., de Gouw J.A. (2017). Emissions of volatile organic compounds (VOCs) from concentrated animal feeding operations (CAFOs): Chemical compositions and separation of sources. Atmos. Chem. Phys. Discuss..

[6] Edwards D., Daniel T. (1992). Environmental impacts of on-farm poultry waste disposal—A review. Bioresour. Technol..

[7] Osterberg D., Wallinga D. (2004). Addressing Externalities From Swine Production to Reduce Public Health and Environmental Impacts. Am. J. Public Health.

[8] Copeland C. (2011). Animal Waste and Water Quality: EPA’s Response to the Waterkeeper Alliance Court Decision on Regulation of CAFOs.

[9] US EPA (2005). Protecting Water Quality from Agricultural Runoff.

[10] US EPA (2004). Risk Assessment Evaluation for Concentrated Feeding Operations.

[11] Graham J.P., Nachman K.E. (2010). Managing waste from confined animal feeding operations in the United States: The need for sanitary reform. J. Water Health.

[12] Wilson S.M., Serre M.L. (2007). Examination of atmospheric ammonia levels near hog CAFOs, homes, and schools in Eastern North Carolina. Atmos. Environ..

[13] Wilson S.M., Serre M.L. (2007). Use of passive samplers to measure atmospheric ammonia levels in a high-density industrial hog farm area of eastern North Carolina. Atmos. Environ..

[14] Camillo N.G.D. (2011). Methane Digesters and Biogas Recovery—Masking the Environmental Consequences of Industrial Concentrated Livestock Production. J. Environ. Law.

[15] US EPA (2015). Chemical Contaminant Rules [Other Policies and Guidance].

[16] Sahoo P.K., Kim K., Powell M.A. (2016). Managing Groundwater Nitrate Contamination from Livestock Farms: Implication for Nitrate Management Guidelines. Curr. Pollut. Rep..

[17] Donham K.J., Wing S., Osterberg D., Flora J.L., Hodne C., Thu K.M., Thorne P.S. (2007). Community Health and Socioeconomic Issues Surrounding Concentrated Animal Feeding Operations. Environ. Health Perspect..

[18] Wing S., Cole D., Grant G. (2000). Environmental Injustice in North Carolina’s Hog Industry. Environ. Health Perspect..

[19] Greger M., Koneswaran G. (2010). The Public Health Impacts of Concentrated Animal Feeding Operations on Local Communities. Fam. Community Health.

[20] Wing S., Wolf S. (2000). Intensive livestock operations, health, and quality of life among eastern North Carolina residents. Environ. Health Perspect..

[21] Radon K. (2006). The two sides of the “endotoxin coin”. Occup. Environ. Med..

[22] Hatcher S.M., Rhodes S.M., Stewart J.R., Silbergeld E., Pisanic N., Larsen J., Jiang S., Krosche A., Hall D., Carroll K.C. (2017). The prevalence of antibiotic-resistant Staphylococcus aureus nasal carriage among industrial hog operation workers, community residents, and children living in their households: North Carolina, USA. Environ. Health Perspect..

[23] Rinsky J.L., Nadimpalli M., Wing S., Hall D., Baron D., Price L.B., Larsen J., Stegger M., Stewart J., Heaney C.D. (2013). Livestock-Associated Methicillin and Multidrug Resistant Staphylococcus aureus Is Present among Industrial, Not Antibiotic-Free Livestock Operation Workers in North Carolina. PLoS ONE.

[24] Abara W., Wilson S.M., Burwell K. (2012). Environmental Justice and Infectious Disease: Gaps, Issues, and Research Needs. Environ. Justice.

[25] Guidry V.T., Rhodes S.M., Woods C.G., Hall D.J., Rinsky J.L. (2018). Connecting Environmental Justice and Community Health: Effects of Hog Production in North Carolina. N. C. Med. J..

[26] Nicole W. (2013). CAFOs and Environmental Justice: The Case of North Carolina. Environ. Health Perspect..

[27] Wilson S.M., Howell F., Wing S., Sobsey M. (2002). Environmental injustice and the Mississippi hog industry. Environ. Health Perspect..

[28] Son J.-Y., Muenich R.L., Schaffer-Smith D., Miranda M.L., Bell M.L. (2021). Distribution of environmental justice metrics for exposure to CAFOs in North Carolina, USA. Environ. Res..

[29] Lenhardt J., Ogneva-Himmelberger Y. (2013). Environmental Injustice in the Spatial Distribution of Concentrated Animal Feeding Operations in Ohio. Environ. Justice.

[30] Ogneva-Himmelberger Y., Huang L., Xin H. (2015). CALPUFF and CAFOs: Air Pollution Modeling and Environmental Justice Analysis in the North Carolina Hog Industry. ISPRS Int. J. Geo-Inf..

[31] Walljasper C. (2018). Large Animal Feeding Operations on the Rise. Investigate Midwest. https://investigatemidwest.org/2018/06/07/large-animal-feeding-operations-on-the-rise/.

[32] [Dataset] Maisenholder, Karen, Census Tracts 2010, Maryland GIS Data Catalog: Demographics, 2010. https://data.imap.maryland.gov/datasets/maryland-census-data-census-tracts.

[33] [Dataset] American Community Survey Tables: 2014–2018 (5-Year Estimates), U.S. Census Bureau Prepared by Social Explorer. 2019, DP05 Table. https://data.census.gov/cedsci/table?q=acs%202018&tid=ACSDP5Y2018.DP05&y=2018&vintage=2018&hidePreview=false.

[34] Nicolaides B., Wiese A. (2017). Suburbanization in the United States after 1945. Oxford Research Encyclopedia of American History.

[35] (2018). The American Community Survey. https://www.esri.com/content/dam/esrisites/en-us/media/whitepaper/WF2012-j10020-american-community-survey-2018-rev.pdf.

[36] [Dataset] Meat, Poultry and Egg Product Inspection Directory. https://www.fsis.usda.gov/wps/portal/fsis/topics/inspection/mpi-directory.

[37] Zboreak V. (2015). “Yes, in Your Backyard!” Model Legislative Efforts to Prevent Communities from Excluding CAFOs.

[38] Bernhardt C., Burkhart K., Schaeffer E. (2015). More Phosphorus, Less Monitoring, Environmental Integrity Project. http://environmentalintegrity.org/wp-content/uploads/Poultry-report_2013_FINAL1.pdf.

[39] United States Census Bureau (2018). American Community Survey 5-Year Data (2009–2018). The United States Census Bureau. https://www.census.gov/data/developers/data-sets/acs-5year.html.

[40] Jacques M.L., Gibbs C., Rivers L., Dobson T. (2012). Expanding Environmental Justice: A Case Study of Community Risk and Benefit Perceptions of Industrial Animal Farming Operations. Race Gend. Cl..

[41] Pendall R., Goodman L., Zhu J., Gold A. (2016). The Future of Rural Housing.

[42] Schultz A.A., Peppard P., Gangnon R.E., Malecki K.M. (2019). Residential proximity to concentrated animal feeding operations and allergic and respiratory disease. Environ. Int..

[43] Cox J. (2020). For Communities of Color, Air Pollution May Heighten Coronavirus Threat. Bay J..

[44] Artz G.M., Orazem P.F., Otto D.M. (2007). Measuring the Impact of Meat Packing and Processing Facilities in Nonmetropolitan Counties: A Difference-in-Differences Approach. Am. J. Agric. Econ..

[45] Artz G.M. (2012). Immigration and Meatpacking in the Midwest. Choices.

[46] Broadway M. (2007). Meatpacking and the Transformation of Rural Communities: A Comparison of Brooks, Alberta and Garden City, Kansas. Rural. Sociol..

[47] Dalla R.L., Christensen A. (2005). Latino Immigrants Describe Residence in Rural Midwestern Meatpacking Communities: A Longitudinal Assessment of Social and Economic Change. Hisp. J. Behav. Sci..

[48] Taitan P.A., McTarnaghan S., Arena O., Su Y. (2018). State of Immigrants in the District of Columbia, Urban Institute. https://www.urban.org/sites/default/files/publication/99031/state_of_immigrants_in_dc_brief_2.pdf.

[49] Frey W.H. (2011). Melting Pot Cities and Suburbs: Racial and Ethnic Change in Metro America in the 2000s, Metropolitan Policy Programs. https://www.brookings.edu/wp-content/uploads/2016/06/0504_census_ethnicity_frey.pdf.

[50] Iceland J., Steinmetz E. (2003). “The Effects of Using Census Block Groups Instead of Census Tracts When Examining Residential Housing Patterns”. U.S. Census Bureau Working Paper. http://www.census.gov/hhes/www/housing/resseg/pdf/unit_of_analysis.pdf.

[51] Animal Feeding Operations (AFOS). https://mde.maryland.gov/programs/LAND/RecyclingandOperationsprogram/Pages/AFOInfo.aspx.

[52] Bullard R.D., Johnson G.S. (2000). Environmentalism and Public Policy: Environmental Justice: Grassroots Activism and Its Impact on Public Policy Decision Making. J. Soc. Issues.

[53] Lockhart K., King A., Harter T. (2013). Identifying sources of groundwater nitrate contamination in a large alluvial groundwater basin with highly diversified intensive agricultural production. J. Contam. Hydrol..

[54] Mirabelli M.C., Zock J.-P., Plana E., Antó J.M., Benke G., Blanc P.D., Dahlman-Höglund A., Jarvis D., Kromhout H., Lillienberg L. (2007). Occupational risk factors for asthma among nurses and related healthcare professionals in an international study. Occup. Environ. Med..

[55] Nadimpalli M., Rinsky J.L., Wing S., Hall D., Stewart J., Larsen J., Nachman K.E., Love D.C., Pierce E., Pisanic N. (2015). Persistence of livestock-associated antibiotic-resistantStaphylococcus aureusamong industrial hog operation workers in North Carolina over 14 days. Occup. Environ. Med..

[56] (2020). Community Healthy Air Act.

[57] USDA/NASS (2019). State Agriculture Overview for Maryland. https://www.nass.usda.gov/Quick_Stats/Ag_Overview/stateOverview.php?state=MARYLAND.

